# Relating surface roughness and magnetic domain structure to giant magneto-impedance of Co-rich melt-extracted microwires

**DOI:** 10.1038/srep46253

**Published:** 2017-04-11

**Authors:** S. D. Jiang, T. Eggers, O. Thiabgoh, D. W. Xing, W. D. Fei, H. X. Shen, J. S. Liu, J. R. Zhang, W. B. Fang, J. F. Sun, H. Srikanth, M. H. Phan

**Affiliations:** 1 School of Materials Science and Engineering, Harbin Institute of Technology, Harbin 150001, P. R. China; 2 Department of Physics, University of South Florida, Tampa, FL 33620, USA; 3 School of Materials Science and Engineering, Inner Mongolia University of Technology, Hohhot 010051, P. R. China; 4 School of Materials Science and Engineering, Harbin University of Science and Technology, Harbin 150080, P. R. China

## Abstract

Understanding the relationship between the surface conditions and giant magneto-impedance (GMI) in Co-rich melt-extracted microwires is key to optimizing their magnetic responses for magnetic sensor applications. The surface magnetic domain structure (SMDS) parameters of ~45 μm diameter Co_69.25_Fe_4.25_Si_13_B_13.5-x_Zr_x_ (*x* = 0, 1, 2, 3) microwires, including the magnetic domain period (*d*) and surface roughness (*R*_q_) as extracted from the magnetic force microscopy (MFM) images, have been correlated with GMI in the range 1–1000 MHz. It was found that substitution of B with 1 at. % Zr increased *d* of the base alloy from 729 to 740 nm while retaining *R*_q_ from ~1 nm to ~3 nm. A tremendous impact on the GMI ratio was found, increasing the ratio from ~360% to ~490% at an operating frequency of 40 MHz. Further substitution with Zr decreased the high frequency GMI ratio, which can be understood by the significant increase in surface roughness evident by force microscopy. This study demonstrates the application of the domain period and surface roughness found by force microscopy to the interpretation of the GMI in Co-rich microwires.

Co-rich melt-extracted amorphous microwires have been recognized as an exciting magnetic material involved in various applications^1^,^2^,^3^,^4^,^5^, including geomagnetic detection sensors^6^, magnetic induction hyperthermia^7^,^8^, and detection of magnetically labeled biomolecules^9^. Amorphous microwires possess super soft magnetic properties that exhibit fascinating magnetic phenomena such as the giant magneto-impedance (GMI) effect^10^,^11^. As it is well known, the longitudinal dc magnetization process of Co-rich amorphous microwires, especially near zero magnetic field, is mainly determined by domain wall movement and rotational magnetization. The latter mechanism is closely related to high frequency magneto-impedance, since at frequencies below ~10 MHz, eddy currents damp domain wall motion^11^. Both processes, however, are closely associated with the surface magnetic domain structure (SMDS), which is essential for interpreting the GMI effect at high frequencies.

Recently, there has been some discussion regarding the stability or presence of the so-called bamboo surface domain structure in negative magnetostrictive Co-based microwires^12^,^13^. In current literature, magneto-optical methods have detected such a SMDS in magnetic microwires^14^,^15^,^16^. One method is via magneto-optical Kerr effect (MOKE) microscopy^17^, which is an optical technique that measures the change in polarization of light after it is reflected from the surface of a magnetic material. In addition, the Faraday effect has been used to image magnetic domains in wires through a technique called the magneto-optical indicator film (MOIF) method^18^,^19^ However, there have been some magnetoimpedance and EMF measurements^20^,^21^ and theoretical calculations^22^ with implications that the bamboo SMDS is metastable, i.e., not present in a ‘perfect’ negative magnetostrictive microwire (and that the SMDS of such a wire is single domain), and that the origin of a bamboo SMDS could be due to a surface anisotropy induced by surface irregularities^22^,^23^.

Since its development from atomic force microscopy (AFM) in 1987^24^, magnetic force microscopy (MFM) has been accepted as an effective technique in detecting surface micro-magnetic information^25^,^26^,^27^. Consequently, MFM images have also detected the presence of a bamboo domain structure in Co-rich microwires^28^,^29^,^30^,^31^,^32^,^33^ while AFM has been used to detect surface defects and deformations. However, there has been no attempt to quantitatively correlate the observed surface roughness and bamboo domain structure with GMI measurements. In this work, we relate the high frequency longitudinal GMI measurements to the surface roughness and bamboo SMDS period imaged by AFM and MFM. The correlation of these measurements on Zr-substituted Co_68.15_Fe_4.35_Si_12.25_B_15.25-x_Zr_x_ microwires (*x* = 0–3) has allowed us to address this issue, while also giving good guidance on optimization of the high frequency GMI responses of Co-rich microwires for advanced sensor applications.

## Results and Discussion

### Magnetic Force Microscopy (MFM) Image Processing

**Figure 1:**
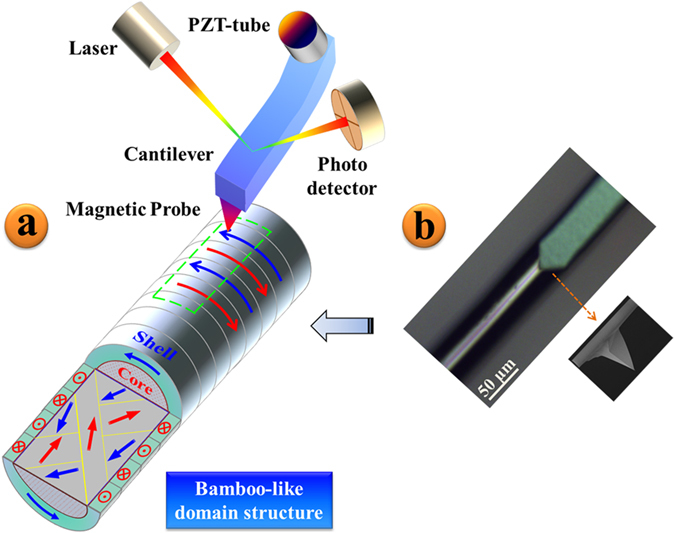
Schematic diagram (**a**) and optical image capture (**b**) of the MFM process. The theoretical “Core-Shell” and surface “Bamboo-like” domain structures are shown in (**a**).

**Figure 2:**
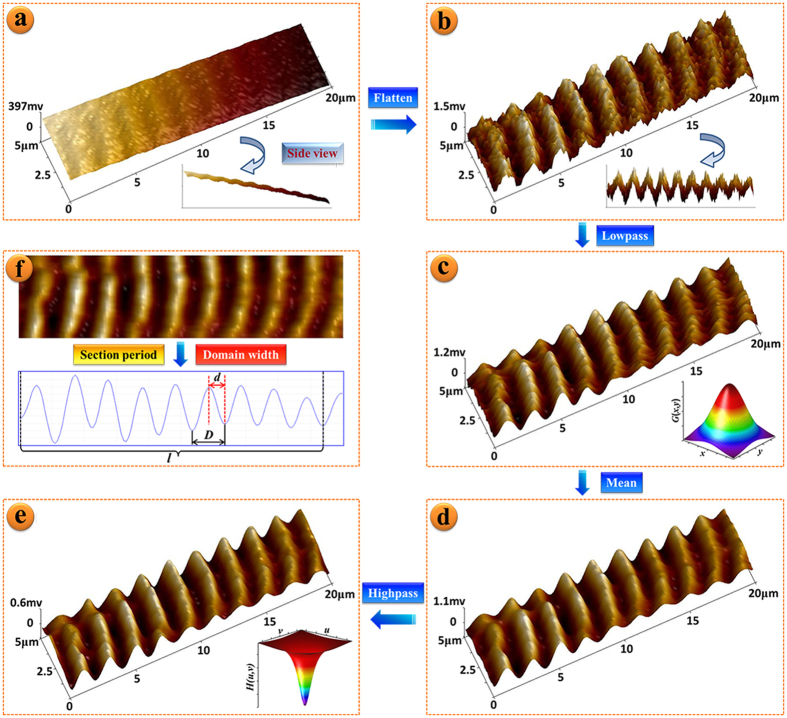
Flow chart of MFM image processing including (**a**) original image, (**b**) Flatten (linear background subtraction), (**c**) Gaussian-Lowpass, (**d**) Mean and (**e**) Butterworth-Highpass filter processing. Inset of (**a**) and (**b**) are side view of 3D-MFM images, inset of (**c**) and (**e**) are the lowpass and highpass function images, respectively, and the inset of (**f**) shows the 2D-MFM image and section period drawing.

Soft magnetic materials can be imaged with great sensitivity using CoCr thin film-coated silicon tips with Q values between 100–200^34^. An MFM image is measured by mapping the varying resonance condition of a cantilever with a perpendicularly magnetized tip during its interaction with the normal component of the stray field of the magnetic material, as seen in Fig. 1(a). It was found that the image of the amplitude and phase change of the cantilever gave identical features, with the former providing a greater contrast. The average value of the magnetic field near the probe is measured instead of discrete values and it shows the negligible influences on the MFM image when the lift height is larger than the size of probe. In this study, the microwire sample was fixed to a horizontal substrate before it was placed under a CoCr-coated silicon probe as shown in a top down view in Fig. 1(b). The tip profile is shown as an inset in Fig. 1(b), with a tip height of 15 ± 2 μm, radius of 50 ± 5 nm, and force constant of 3 N/m. The MFM image is 128 px × 32 px and scanning rate is 0.996 Hz. Figure 2(a–f) illustrates the image processing steps taken to transform an initial 3D MFM image of a Co_68.15_Fe_4.35_Si_12.25_B_14.25_Nb_1_ microwire into the restored image used to extract the surface domain period, *d*. In an effort to be transparent with our method of determining the domain period, we chose the Co_68.15_Fe_4.35_Si_12.25_B_14.25_Nb_1_ microwire since it had a very uniform domains structure. In the following section, the details of the image processing steps are given.

Over the course of a long scan, thermal and mechanical induced drift affects the MFM image by imposing a linear background as shown in the side view of Fig. 2(a). This image becomes normal after the linear background is fit and subtracted out (side view of Fig. 2(b)). Since the signal of surface stray field is mainly in the very low frequency region, high frequency noise present in the image can be removed without affecting the magnetic data. Therefore, a Gaussian-Lowpass filter was applied to smooth Fig. 2(b) in order to enhance the signal to noise ratio (SNR), which ultimately aids in the extraction of the magnetic parameter *d*. Since the Gaussian transfer function (illustrated in the inset of Fig. 2(c)) is a single-valued function with rotational symmetry, the local characteristics of the image edge are not distorted by the process. Figure 2(c) shows stripe-like noise along the wire axis from the scanning pattern of the probe still present in the image. To reduce the contribution from the stripe noise to the parameter extraction, a mean, or convolution, filter is used to further smooth Fig. 2(c) and the results are shown in Fig. 2(d). The size of the discrete convolution sliding window was chosen to be 5 px × 5 px or 0.609 μm^2^. In order to restore detail to image features that were lost by application of the mean filter, a high-pass filter is applied. The final image used for parameter extraction is shown in Fig. 2(e).

### Surface Magnetic Domain Structure (SMDS) Analysis

**Figure 3:**
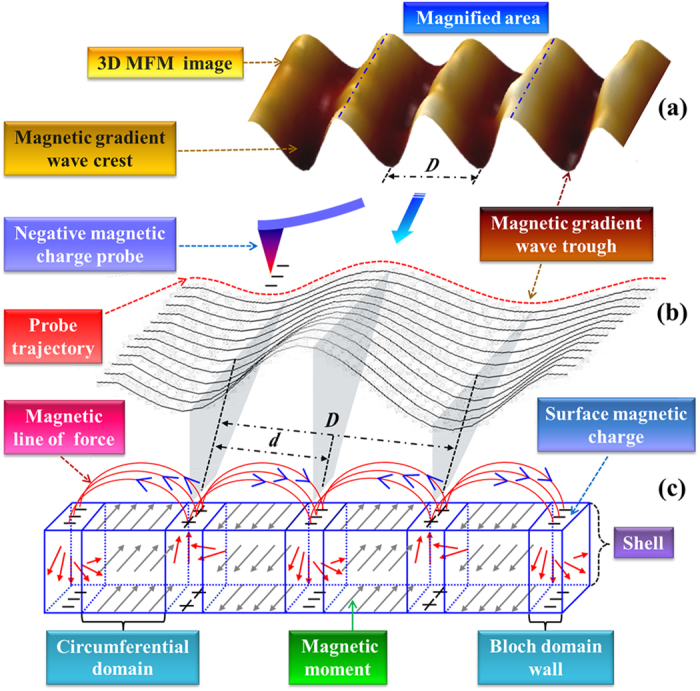
Model and analysis chart of the relation between 3D-MFM image and SMDS; (**a**) is a 3D-MFM image, (**b**) shows a diagram of the chosen area in (**a**) and the red curve is an example of an MFM scanline, (**c**) presents the core-shell model with circumferential domain and Bloch wall structures of the outer shell in Co-rich amorphous microwires.

The final processed image is analyzed and connected to a theoretical model of magnetic domains to understand the observed periodicity of the SMDS of the Co-rich microwires. According to the core-shell model of negative magnetostrictive Co-rich amorphous microwires^11^, the magnetic moments of the surface domains are nearly aligned in the circumferential direction. As one can see in Fig. 3 (a–c), there are two potential sources of surface stray fields according to this picture^35^: one is generated by the domain magnetization and the other by the domain wall. Consequently, there is no stray field in the normal direction due to domain magnetization. However, the Bloch wall has a stray field normal component that is detected by MFM. The stray field from the Bloch walls will generate a magnetic flux leakage field and thus the surface position of the domain wall can be determined by these intense magnetic field lines. The basis for the contrast in the MFM image is that positive (negative) magnetic charges produced at a surface/domain wall interface interacts with the MFM probe in the following way: The probe is magnetized to be in a negative remanent state, such that the magnetic force (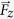) gradient along the *z*-axis is a minimum if the surface stray field flows out of the surface normal. This corresponds to the wave trough as shown in Fig. 3 (a–c). The wave crest corresponds to a repulsive force, where 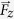 is a maximum. However, based on the above analysis, it is not possible to estimate either the width of the domain or domain wall; only the combined width of the domain and wall can be determined by extracting the average period from crest-to-crest or trough-to-trough, marked as *D* in Fig. 3(a). We can consider *d* = *D*/2 to represent the domain period as shown in Fig. 3(b).

### Effects of Zr substitution on the surface roughness, SMDS, and GMI

**Figure 4:**
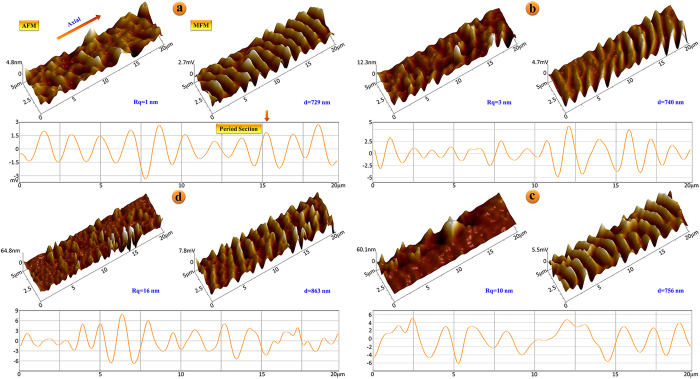
3D AFM and MFM images of (**a**) Zr0, (**b**) Zr1, (**c**) Zr2, and (**d**) Zr3-doped microwires. Below each pair of images is a single MFM scanline.

AFM and MFM measurements on a series of Co_68.15_Fe_4.35_Si_12.25_B_15.25-x_Zr_x_ microwires were conducted as a function of transition metal Zr substitution (*x* = 0–3). Figure 4(a–d) shows 3D AFM and MFM images of the Zr-doped wires along with one MFM scanline down the wire axis used to determine the average value of the domain period, *d*. For the base composition of wire (*x* = 0), the average surface roughness and domain period were found to be *R*_*q*_ = 1.14 nm and *d* = 729 nm, respectively. With increasing Zr content, both surface roughness and domain period increased with *R*_*q*_ = 3.29 nm and *d* = 740 nm for Zr_1_; *R*_*q*_ = 9.86 nm and *d* = 756 nm for Zr_2_; and *R*_*q*_ = 15.6 nm and *d* = 863 nm for Zr_3_.

**Figure 5:**
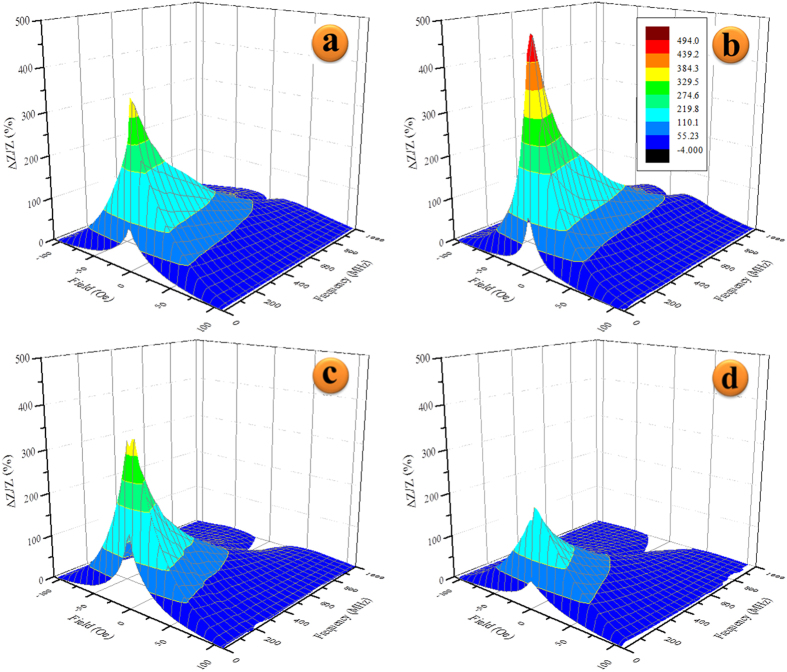
Evolution of the GMI effect in Co_68.15_Fe_4.35_Si_12.25_B_15.25-x_Zr_x_ microwires with (**a**) *x* = 0, (**b**) *x* = 1, (**c**) *x* = 2, and (**d**) *x* = 3 as a function of magnetic field and frequency (1–1000 MHz).

**Figure 6:**
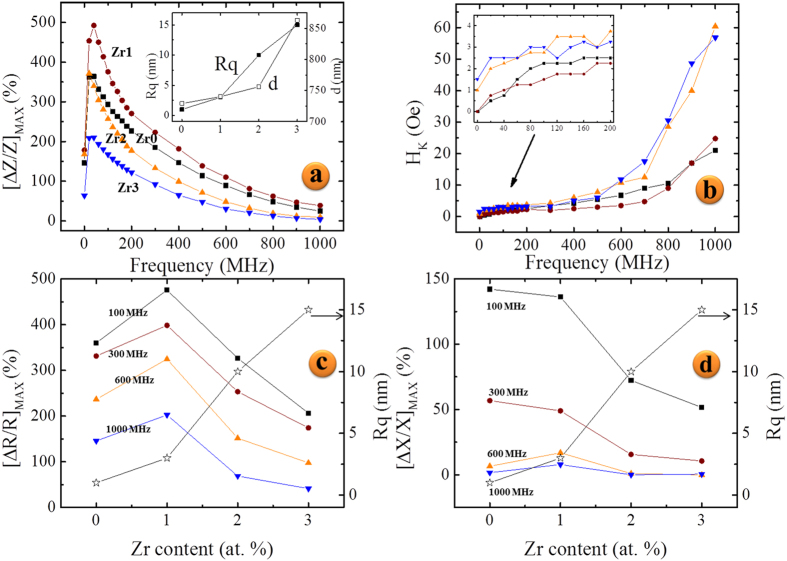
Maximum change in impedance (**a**), anisotropy field (**b**), maximum change in resistance of Co_68.15_Fe_4.35_Si_12.25_B_15.25-x_Zr_x_ microwires with *x* = 0–3 denoted by square, circle, triangle, and inverted triangle symbols, respectively. Inset of (**a**) shows the correlation of average surface roughness *R*_*q*_ with domain period *d*. Inset of (**b**) shows zoomed in portion. (**c**) The maximum change in resistance and (**d**) the maximum change in reactance as a function of Zr content at selected frequencies. The right axis of (**c**,**d**) is the domain period denoted by star symbol.

Magnetoimpedance measurements at the wide frequency range of 1–1000 MHz were conducted on all the microwires. Figure 5(a–d) shows the field and frequency evolution of the change in impedance, Δ*Z/Z*, for the microwires with 0, 1, 2, and 3 at. % Zr, respectively. The 1 at. % Zr substitution shows a beneficial increase of Δ*Z/Z* ~490% at a frequency of 40 MHz over the other compositions. Further substitution past 1 at. % suppresses the Δ*Z/Z* ratio at higher frequencies, as seen by the flat regions in dark blue in Fig. 5(c) and (d). Figure 6(a) presents the maximum change in impedance, [Δ*Z/Z*]_max_, of the base composition and Zr-doped wires over the measured frequency range. It is apparent that 1 at. % Zr substitution boosts the maximum impedance change over the whole frequency range while any further substitution of Zr brings [Δ*Z/Z*]_max_ down to and below *x* = 0, depending on the excitation frequency. At frequencies below ~140 MHz, [Δ*Z/Z*]_max_ of *x* = 2 is roughly similar to the *x* = 0 wire, while at higher frequencies [Δ*Z/Z*]_max_ values drop steadily lower.

The DC magnetic field also modifies the skin depth through the circumferential (AC) permeability. Since the circumferential permeability is at its highest value at *H*_*DC*_ = *H*_*K*_, the skin depth is a minimum at this field for any given frequency. At *H*_DC_ = *H*_max_, the circumferential permeability is at its lowest value and the skin depth is a maximum. A simple model can be used to determine the penetration depth of the skin effect based on the assumption that the real part of the impedance change is due only to changes in effective cross-sectional area^36^. With this assumption, Fig. 6(c) can represent not only the maximum change in resistance, [ΔR/R]_max_, but also the maximum change in skin depth at any given frequency. Figure 6(d) presents the maximum change in reactance, [ΔX/X]_max_, for the microwires. This component of the impedance is generally analyzed through inductance formalism, where the imaginary part of the impedance undergoes a simple geometrical transformation to the real part of the inductance^37^. The real part of the inductance is then proportional to the real part of the complex permeability, which represents conservative magnetization processes such as reversible magnetization rotation.

## Discussion

Energetically, one can determine the equilibrium domain period based on minimization of the magnetostatic energy and domain wall energy contributions as done in ref. 22. The magnetostatic contribution depends on wire radius and cross-sectional shape, while the domain wall energy term depends on the thickness of the outer shell and anisotropy and exchange constants. With increasing shell thickness and ellipticity of wire cross section, the domain period is found to increase, while the domain period decreases with increasing surface anisotropy. We consider the substitution of transition metal Zr for B to mainly affect the stress induced during fabrication by removal of the amorphous component B and introducing more crystallite nucleation sites with Zr^4^, since all fabrication parameters were held constant for all the microwires. This could have the impact of increasing/decreasing the thickness of the outer shell, inducing irregular surface morphologies, and/or affecting the induced surface anisotropy. This seems a reasonable assumption considering the positive correlation of the average surface roughness and domain period in the wires, as seen in the inset of Fig. 6(a).

Figure 6(a,b) shows that substitution with *x* = 1 at. % has the highest [Δ*Z/Z*]_max_ and lowest *H*_*K*_ over the majority of the spectrum. Considering this, it can be concluded that the slight increase of domain period in the *x* = 1 at. % could originate from a reduction of induced surface anisotropy. With Zr substitution of 2–3 at. %, the *H*_*K*_ increases dramatically over the whole frequency range especially at high frequencies where the penetration depth is close to the surface. This might suggest an increase in induced surface anisotropy, however the domain period at the surface is observed to increase as well. It is likely then that the anisotropy is not responsible for the domain period increase in this case, and the observed roughness increase by a factor of 3 could point to a deformity in wire cross section or an increase in shell thickness, since these factors can increase the domain period^22^.

Figure 6(c,d) show that the resistance is the major contributor to the impedance over all frequencies, while the maximum change in reactance drops to near zero percent at *f* > 600 MHz. This behavior is expected at this frequency range as the experimental conditions approach that of ferromagnetic resonance, where the real part of the impedance continues to climb until resonance is reached while the imaginary part approaches zero. However, the near zero *percent change* in the reactance is simply a consequence of the definition (Eq. 2); the applied field at which the reactance is a maximum for *f* > 600 MHz has shifted beyond *H*_*max*_ = 110 Oe.

It is apparent that the reduction of [Δ*Z/Z*]_max_ as Zr substitution is increased to 2 and 3 at. % is greatly affected by the surface roughness increase, which plays a crucial role at high frequencies where the skin depth is small^38^,^39^. The large *H*_*K*_ and wide field-dependent GMI curves (Fig. 5(c,d)) at frequencies greater than 600 MHz indicate a much larger surface anisotropy distribution than *x* = 0 or 1%. This suggests an increase in fluctuations of local magnetic anisotropy and saturation magnetization at the surface and is well known to negatively impact [Δ*Z/Z*]_max_ along with surface defects that can immobilize surface spins and reduce the GMI ratio^23^,^40^.

In summary, the Zr-substitution of B in Co_68.15_Fe_4.35_Si_12.25_B_15.25-x_Zr_x_ microwires was conducted to establish the relationship between the surface roughness, MFM and the GMI effect. Both AFM and MFM images were observed and standard image processing techniques were used to extract surface magnetic parameters meaningful to the analysis of the GMI effect. By linking the observed MFM to the core-shell model of negative magnetostrictive microwires, the magnetic domain period could be established. Coupled with the average surface roughness and qualitative observations, AFM/MFM images are significant tools capable of quantitative analysis of the influences of domain structure changes on the GMI effect, especially changes which are subtly induced by chemical substitution. Our study provides a new pathway for producing Co-rich amorphous microwires with desired GMI response for advanced sensor applications.

## Methods

### Sample Preparation

High-quality Co_68.15_Fe_4.35_Si_12.25_B_15.25-x_Zr_x_ (*x* = 0–3) amorphous microwires with diameter of ~45 μm and ~50 mm in length were fabricated by melt-extraction described elsewhere^30^,^33^.

### Properties Characterization

The magnetic force microscopy in this work was conducted on a Bruker Dimension Icon in tapping-lift mode with the cantilever driven slightly below its natural resonance frequency to maximize the change in oscillation amplitude. The lift height was kept constant at 100 nm for all samples.

Magneto-impedance was measured as a function of applied magnetic field (*H*_*max*_ = 110 Oe) along the length of the wire at high frequency (1–1000 MHz) using an HP 4191A impedance analyzer. The HP 4191A determines the complex reflection coefficient, Γ, of a high frequency signal sent down a terminated transmission line. An airline^41^ made of a 2 cm section of wire suspended over a Cu ground plane served as the transmission line, which was terminated with a 50 Ω standard that matches the input impedance of the analyzer. The complex reflection coefficient at the beginning of the microwire airline was measured and converted to complex impedance (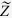) by


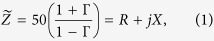


where *R* is the resistance, *X* is the reactance, and *j* is the imaginary unit. In this work, the magnetoimpedance ratio is defined as


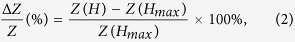


where *Z(H*) is the impedance (or resistance, reactance) at field *H.* The impedance ratio is normalized by *Z(H* = *H*_*max*_). The *H*_DC_ location of the maximum change in impedance is denoted by *H*_K_, which is typically referred to as the anisotropy field.

## Additional Information

**How to cite this article**: Jiang, S. D. *et al*. Relating surface roughness and magnetic domain structure to giant magneto-impedance of Co-rich melt-extracted microwires. *Sci. Rep.* **7**, 46253; doi: 10.1038/srep46253 (2017).

**Publisher's note:** Springer Nature remains neutral with regard to jurisdictional claims in published maps and institutional affiliations.
